# Predictive multiphase evolution in Al-containing high-entropy alloys

**DOI:** 10.1038/s41467-018-06757-2

**Published:** 2018-10-30

**Authors:** L. J. Santodonato, P. K. Liaw, R. R. Unocic, H. Bei, J. R. Morris

**Affiliations:** 1 Oak Ridge National Laboratory, Oak Ridge, TN 37831 USA; 2 Department of Materials Science and Engineering, The University of Tennessee, Knoxville, TN 37996 USA; 3 Advanced Research Systems, Macungie, PA 18018 USA

## Abstract

The ability to predict and understand phases in high-entropy alloys (HEAs) is still being debated, and primarily true predictive capabilities derive from the known thermodynamics of materials. The present work demonstrates that prior work using high-throughput first-principles calculations may be further utilized to provide direct insight into the temperature- and composition-dependent phase evolution in HEAs, particularly Al-containing HEAs with a strengthening multiphase microstructure. Using a simple model with parameters derived from first-principles calculations, we reproduce the major features associated with Al-containing phases, demonstrating a generalizable approach for exploring potential phase evolution where little experimental data exists. Neutron scattering, in situ microscopy, and calorimetry measurements suggest that our high-throughput Monte Carlo technique captures both qualitative and quantitative features for both intermetallic phase formation and microstructure evolution at lower temperatures. This study provides a simple approach to guide HEA development, including ordered multi-phase HEAs, which may prove valuable for structural applications.

## Abstract (web summary)

Exploration of high entropy alloy phases where little experimental data exists is still challenging. Here, the authors develop an approach where parameters from first principle simulations are incorporated into Monte Carlo simulations to reproduce phase evolution of aluminium-containing high entropy alloys.

## Introduction

The fundamental nature of structural order-disorder transitions in binary alloys was explained in the early twentieth century, in large part due to the work of Bragg and Williams, by considering only the nearest-neighbor interaction energies and the configurational entropy of atoms populating a fixed lattice^1^. The present work uses a similar approach to model ordering and related cooling transformations, which often occur in multi-element alloys, such as high-entropy alloys (HEAs)^2–15^. The present examination of HEA cooling transformations originated in the context of the entropy hypothesis that disordered solid-solution phases will be stabilized in HEAs, due to their high configurational entropy, relative to the competing phases. A recent comprehensive review^4^ has established that enthalpy plays a critical role in determining which compositions form single solid-solution phases with no long-range configurational order. Troparevsky et al. have demonstrated that predicting single-phase compounds may largely be achieved by considering the enthalpy gain of forming ordered phases from each potential binary combination^7,16^. This procedure may be done strictly from high-throughput first-principle calculations, without input from experiments, and largely through a remarkably simple “enthalpy matrix” derived from a large set of calculations.

Clearly, the above approach is one step in a broader fundamental problem: one would like to know not only that a particular composition will show multiple phases, but further what phases would be present at any given temperature, ideally with minimal input from experimental work. To address this challenge, we consider the Al_*x*_TM system, where TM represents combinations of 3*d* transition-metal (TM) elements, such as CoCrFeNi. This system is chosen due to the broad number of studies within the HEA literature^2,10,17–21^, and due to the fact that for sufficient Al, there are a series of phase transformations, including the formation of partially ordered aluminides in a body-centered-cubic (BCC) solid-solution matrix^10,22–24^. By controlling the composition and microstructure through thermal processing associated with the phase transformations, this system provides a wide range of potential compounds and associated microstructures. The strong ordering tendencies of Al with most of the TMs makes the ordering transition of primary importance. As reported in the early literature, some HEAs have room-temperature microstructures of ordered and disordered phase mixtures^2,17^. The Al_*x*_CoCrFeNi alloy forms coherent mixtures of B2 and BCC phases at room temperature^10,17,21,25–27^, particularly when the Al contents are large (*x* > 0.875)^28^. The six-component AlCoCrCuFeNi alloy has a three-phase FCC/BCC/B2 microstructures under most processing conditions, with the face-centered-cubic (FCC) phase being primarily a Cu-based bystander phase that minimally affects the other transformations^17^.

**Fig. 1:**
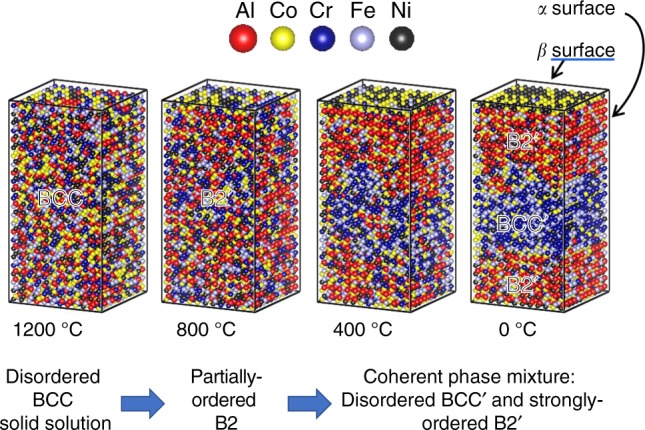
Monte Carlo simulations of a two-step cooling transformation in the AlCoCrFeNi HEA. The atomic distributions for a series of different temperatures are illustrated, using supercells, composed of 12 × 12 × 24 cubic unit cells, where each unit cell contains one *α* and one *β* site. The supercells are “cut” to highlight configurational ordering, such that the side surfaces contain Al-rich *α* sites, and the top surface contains Al-poor *β* sites. Based upon the element-specific long-range order parameters (Fig. 3c), we find that the high-temperature phase is a disordered solid solution, which transitions to a partially ordered phase during cooling to 800 °C. Upon further cooling, the partially ordered phase transforms into a mixture of disordered Cr-Fe-enriched BCC and strongly ordered Al-Co-Ni-enriched B2 phases

Below, we present both calculations and experiments that are consistent with these main transformations in Al_*x*_CoCrFeNi, for 1 < *x* < 2: on cooling from high temperatures (near melting), the high-temperature BCC solid solution transforms into a partially ordered B2 phase. Upon further cooling, the partially ordered B2 phase transforms into a phase mixture comprised of disordered BCC (primarily Co and Fe) plus a B2 phase (primarily composed of Ni-Co-Al). This two-step transformation is illustrated using Monte Carlo simulations (Fig. 1).

## Results

### Monte Carlo model for multi-component transformations

The present Monte Carlo simulations are implemented, using a lattice model, where the atoms are constrained to reside on a fixed lattice—in the present case, a BCC lattice. This model is based on the experimental knowledge of the Al_*x*_CoCrFeNi phases, and represents the only input from experiment. The simulated cooling transformations involve only redistributions of the atomic population, as an approximation to the behavior of real alloys undergoing sublattice ordering and coherent phase separation. This lattice constraint is too restrictive to address all types of HEA transformations, or to provide high-accuracy simulations: it neglects both the possibility of changing the underlying lattice, and also ignores potential vibrational entropy effects associated with small displacements from the average position^29^. However, the lattice-constrained simulations correctly predict the major trends in some well-known HEAs^2,10,17^, especially in their as-cast forms, and provide a quantitative framework for understanding the tendencies to both short- and long-range chemical ordering. The shortcomings are addressed in the Discussion section below.

For simplicity, the interactions are assumed to be near-neighbor only, and solely dependent upon the composition of the near-neighbor sites. The values of the binary interaction energies, *v*_*ij*_, are derived from the enthalpy matrix, *H*_*ij*_, associated with the enthalpy change on ordering from a solid solution, which were recently assembled and applied to the problem of predicting HEA compositions likely to form single-phase solid solutions^7^. The relationship between the *H*_*ij*_ and *v*_*ij*_ is given by

1 $$v_{ij} = \frac{{H_{ij}}}{z}$$

where *z* is the number of the first nearest neighbors in the anticipated HEA structure. This assumption is motivated mainly by the convenience of directly applying the existing *H*_*ij*_ matrix to the calculations. The Monte Carlo moves are the simple exchanges of neighboring atoms, with usual Metropolis acceptance probabilities. The details are specified in the Methods section below.

The values of *v*_*ij*_ are given in Table 1, for the simulations presented, as well as some related components. These values are reasonable, in light of prior experimental observations. First, the interactions between Co-Cr-Fe-Mn-Ni are all small (magnitudes < 2 kJ/mol), indicating the weak interactions in the single-phase forming “Cantor” alloy of CrCrFeMnNi^3^. Second, the addition of Al shows a favorable interaction with every other component, with particular strong interactions with Ni and Co. This trend immediately suggests the formation of a Ni-Co-Al phase at low temperatures, as has been observed^17,21,25^. Roughly speaking, the Co and Ni interactions are reasonably similar, and our results presented below demonstrate that Co and Ni have similar behaviors. Finally, we note that with the exception of weakly attractive interactions with Ni and Al, Cu has a positive interaction with all other components. Thus, we may expect that Cu will tend to segregate away from the others. This trend has been observed experimentally, and is supported by simulations^10,17,30,31^.

**Table 1:** Nearest-neighbor interaction energies, *v*_*ij*_ (kJ/mol)^a^

Elements	Al	Co	Cr	Cu	Fe	Mn	Ni
Al	0	−7.58	−1.66	−1.80	−4.44	−3.35	−8.15
Co	−7.58	0	0.06	0.65	−0.72	−0.23	−0.25
Cr	−1.66	0.06	0	1.30	−0.10	−1.32	−0.36
Cu	−1.80	0.65	1.30	0	0.78	0.35	−0.07
Fe	−4.44	−0.72	−0.10	0.78	0	0.11	−1.17
Mn	−3.35	−0.23	−1.32	0.35	0.11	0	−1.39
Ni	−8.15	−0.25	−0.36	−0.07	−1.17	−1.39	0

^a^Relative to the self-neighbor interactions, based upon formation enthalpies from ref. ^7^

Below, two distinct approaches are used to characterize the development of ordered compounds in the simulated work. The nearest-neighbor pair correlations, *P*_*ij*_, are defined as the average fraction of neighbors to the type-*i* atoms that are type-*j* atoms. The *P*_*ij*_ are normalized such that **Σ**_*j*_ *P*_*ij*_ = 1, and *P*_*ij*_ give the probability that any given nearest neighbor of the element *i* is of the type *j*. In the case of a randomly mixed single phase, the *P*_*ij*_ simply correspond to the fractional concentrations of the potential neighbors, such that *P*_*ij*_ = *x*_*j*_. Deviations from the *P*_*ij*_ = *x*_*j*_ random behavior reveal element-specific ordering, segregation, and phase separation, which are critical issues in the development of HEAs.

**Fig. 2:**
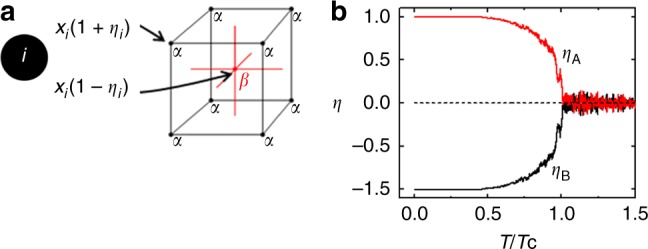
Configurational order parameters. **a** An illustration shows how multiple elements, each with a mole fraction, *x*_*i*_, fill the interpenetrating lattices, *α* and *β*, according to a set of order parameters, *η*_*i*_. **b** In the case of a binary alloy, there is only one independent order parameter, *η* = *η*_A_ = −*η*_B_, which is normally plotted over a range of 0 to 1, and as a function of the reduced temperature, *T*/*T*_c_, where *T*_c_ is the ordering temperature. Here both *η*_A_ and *η*_B_, are plotted, over the range of –1 to 1, as will be necessary for HEAs with four or more principal elements. The plots contain some statistical noise because they were generated from Monte Carlo simulations

The long-range ordering behavior of a single-phase HEA may be expressed, employing a set of parameters, *η*_*i*_, for *i* = 1 to *n* elements. The B2 (CsCl) structure, relevant for the formation of NiAl and other aluminides, may be represented using two interpenetrating sublattices on the BCC lattice, *α* and *β*, with equal numbers of sites, *a*^*α*^ = *a*^*β*^ = 1/2 (Fig. 2a). The fractional occupancy of element *i* on sublattice *α* is denoted as $y_i^\alpha$, and the associated order parameters are given by

2 $$\eta _{i} \, = \, \frac{{y_i^{\alpha} - y_i^{\beta} }} {{y_i^{\alpha} + y_i^{\beta} }}$$

The *η*_*i*_ values fall within the range of −1 to 1, and are related to experimentally observed quantities, such as neutron and X-ray diffraction patterns^10^, as shown below. The case of *η*_*i*_ ≈ 0 indicates that the element, *i*, is found on both sublattices in equal proportions. When *η*_*i*_ approaches positive or negative 1, however, the element is found exclusively on the *α* or *β* sublattice, respectively. The opposite values represent the different possible symmetry-breaking arrangements. For alloys with *n* > 2 elements, the use of multiple order parameters allows an unambiguous description of the atomic-ordering behavior. Each sublattice contains half of the total number of sites, *N*. Vacancies are not considered.

Despite its simplicity, the present model allows us to understand HEA-ordering behaviors in greater depth than the standard classifications based upon binary prototype alloys, such as the B2-ordered CsCl. Although it is correct and useful to classify the HEAs in this fashion, additional information is needed because there are many distinct ways to achieve a given ordering type for compositions of *n* > 2 major elements. The problem may be solved, using sets of element-specific parameters, such as the sublattice occupancies, which have been used in the study of the MoNbTaW alloy^32^, or with order parameters, *η*_*i*_, used here and in earlier work on the Al_1.3_CoCrCuFeNi alloy^10^. Indeed, our work here is similar in spirit to studies on the MoNbTaW alloys^32,33^, though we use a simpler Monte Carlo scheme (utilizing only first neighbors and without the use of molecular dynamics simulations).

### Simulated order-disorder transitions and transformations in HEAs

**Fig. 3:**
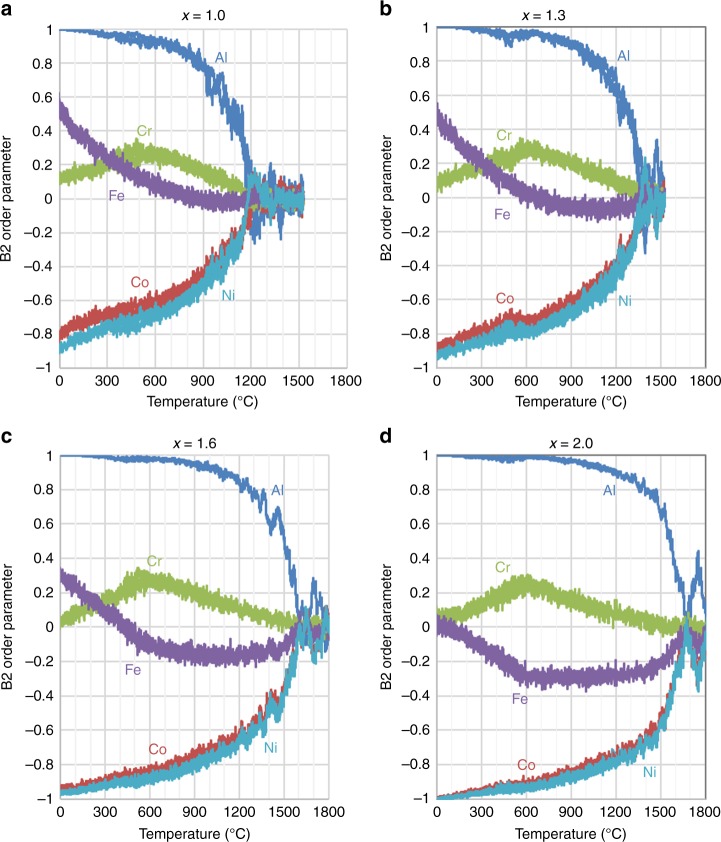
Simulated configurational order. The element-specific B2-order parameters obtained from Monte Carlo simulations are plotted for the Al_*x*_CoCrFeNi family of alloys. A comparison of four different aluminium contents, **a** *x* = 1.0, **b** *x* = 1.3, **c** *x* = 1.6, and **d** *x* = 2.0, shows that the order parameters all tend toward zero above a sufficiently high temperature (i.e., the ordering-onset temperature), which increases with increasing Al content. More importantly, the two distinct behaviors of the strongly ordering (Al, Co, and Ni) and disordered (Cr and Fe) groups of elements, which are seen in all the plots, are crucial to understanding the two-step cooling transformations of the present alloys

All simulated systems are disordered at high temperatures but form an ordered structure as the system is cooled. The progression of structures is shown for *x* = 1 in Fig. 1. To demonstrate this feature more rigorously, and to quantify the development of order, plots of *η*_*i*_ versus temperature from Monte Carlo simulations are shown in Fig. 3, for Al_*x*_CoCrFeNi, with Al compositions ranging from *x* = 1 to *x* = 2. One significant feature of the simulations is that the ordering onset, i.e., where the HEA is cooled below the ordering temperature, *T*_c_, involves the strong ordering of at least two, but not necessarily all, of the *n* > 4 TM elements. The dominant behavior on cooling is that Al segregates to one sublattice, with Ni and Co primarily occupying the other sublattice. All behaviors are quantitatively reproduced on both heating and cooling simulations, indicating that the results are essentially in equilibrium. The effect of the Al content is demonstrated by comparing the Al_2_CoCrFeNi and AlCoCrFeNi HEA systems. In the higher-Al-content Al_2_CoCrFeNi alloy, *T*_c_ is above the experimental melting point, such that the alloy never becomes disordered in the solid phase. Instead, the alloy is partially ordered at the melting temperature. In the lower-Al composition, AlCoCrFeNi, *T*_c_ occurs in the solid phase, near 1200 °C. A more complete description of the B2-ordering trends are discussed below. The fraction of Al-Al pairs is low at all temperatures, indicating that even above the B2-transformation temperature, the Al atoms are primarily pairing with the TMs. Interestingly, Fig. S1 shows that as the Al content increases, the fraction of Al-Al pairs decreases, opposite of what would normally be expected for a disordered material. This trend is due to the stronger tendency to form a B2 phase with increasing Al content. Thus, even in the high temperature, disordered BCC phase, there is significant short-range order around the Al atoms, in contrast to descriptions where the Al is assumed to be randomly organized^34^.

**Fig. 4:**
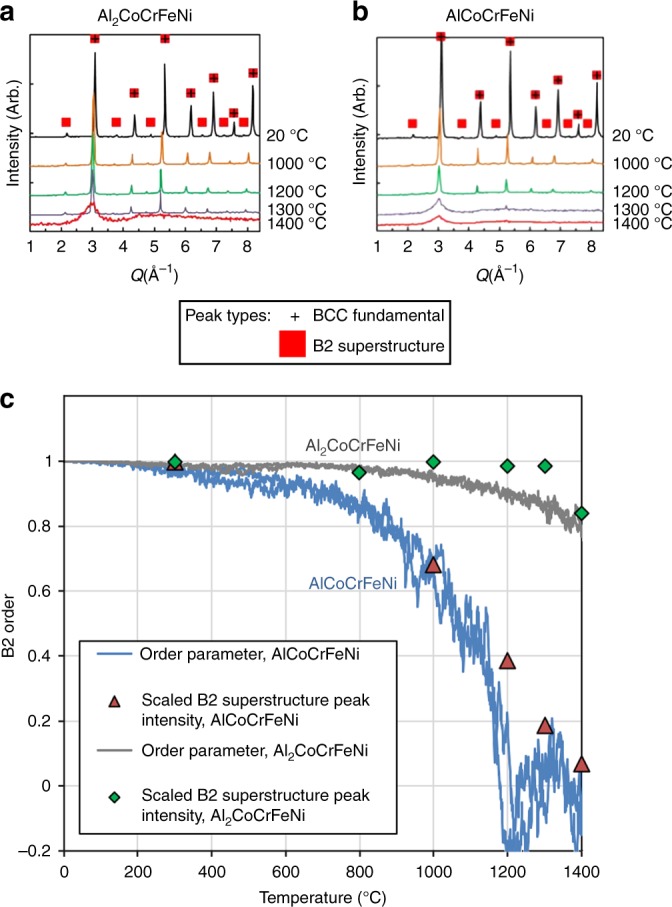
Neutron diffraction patterns from in situ levitated heating studies, compared with simulations. **a **The plots of intensity versus scattering vector magnitude, *Q*, show that the Al_2_CoCrFeNi alloy produces one family of peaks corresponding to the BCC/B2 structure. The B2 superstructure peaks persist until the alloy melts near 1400 °C, indicating that the alloy never reaches the disordered state in the solid phase. **b** The AlCoCrFeNi alloy has a similar BCC/B2 structure. However, the superstructure signal becomes weak during heating, and is not observable at 1200 °C and above, indicating that the alloy may undergo an order-disorder transition. **c** A comparison of the B2 superstructure diffraction peak intensity for *Q* near 2.2 Å^-1^, scaled by the value at 300 °C, with the B2-order parameter, *η*_Al_, from simulations. Note that the highest experimental temperatures shown here are close to the melting transformation. One general feature of all compositions is the peak shift due to thermal expansion

In order to examine the development of B2 order experimentally, in situ neutron scattering studies were conducted from room temperature all the way to the molten phase, using an aerodynamic levitator^35,36^, in a similar fashion to the previous work^10^. The new diffraction patterns of both the Al_2_CoCrFeNi (Fig. 4a) and AlCoCrFeNi (Fig. 4b) demonstrate that the B2/BCC structural evolution proceeds entirely by the rearrangement of atoms on a common BCC lattice, producing a single family of diffraction peaks (i.e., all peaks are indexed to a common lattice parameter) throughout the temperature-dependent phase evolution. These neutron diffraction results highlight the remarkable tendency of the presently studied family of HEAs to form simple structures, even when they undergo ordering and phase separation.

Within the observed family of diffraction peaks, we distinguish between fundamental (common to both the disordered BCC and ordered B2 structures) and superstructure reflections (exclusive to the B2 structure). Both types coexist in ratios that vary with temperature. In the Al_2_CoCrFeNi alloy, the superstructure peaks persist all the way to the melt (Fig. 4a), while in the equimolar AlCoCrFeNi alloy, they are observable only at 1000 °C and below (Fig. 4b). The experimental results in Fig. 4 are consistent with the formation of a partially ordered B2 phase, which occurs directly from the melt in the high-Al-content Al_2_CoCrFeNi alloy, and through an order-disorder transition in the AlCoCrFeNi alloy. To compare with simulations, Fig. 4c shows the superlattice peak intensity, *S*(*Q*), for the peak near 2.2 Å^-1^ for both systems, scaled by their value at *T* = 300 °C. The B2-order parameter, *η*_Al_, from simulations is also shown. The comparison between the diffraction studies and the simulation is strikingly close: both show the loss of the B2 order for AlCoCrFeNi near 1400 °C, while the B2 phase remains strongly ordered at that temperature for Al_2_CoCrFeNi. This trend demonstrates that our simulations quantitatively capture the development of B2 order in these systems.

### Temperature-dependent ordering within the BCC solid solution

**Fig. 5:**
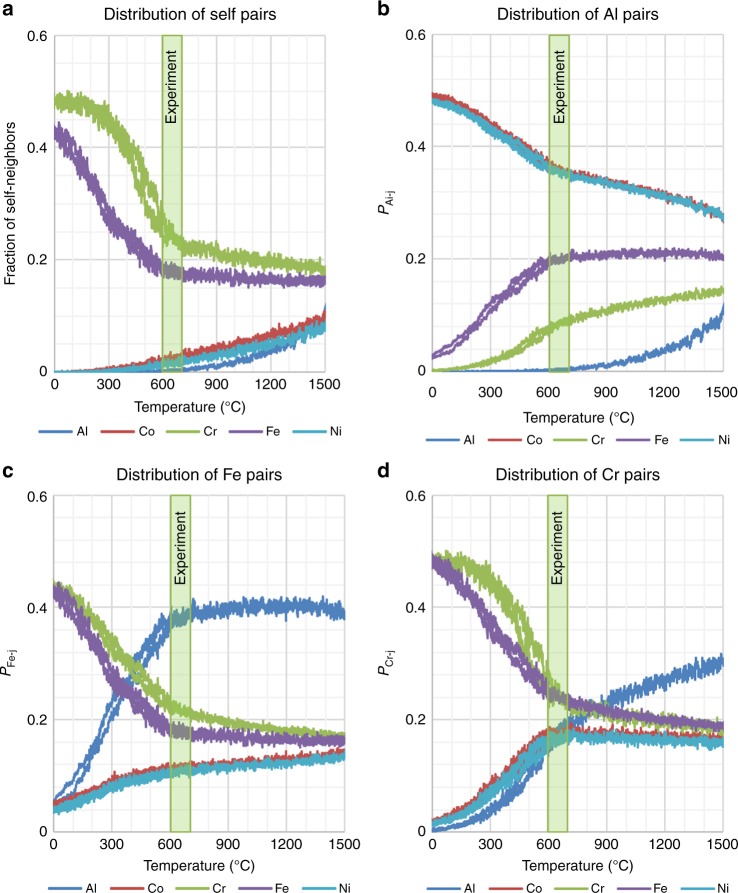
Nearest-neighbor-pair correlations reveal transformations in Al_2_CoCrFeNi. The temperature range where microscopy shows a rapid change in microstructure (600–700 °C; Fig. 6) is highlighted by the shaded box in parts **a**–**d**. **a** The self-pair probabilities, *P*_*ii*_, reveal two groups of elements, the strongly ordering Al, Co, and Ni, with vanishing self-neighbor probability at low temperatures that indicates strong ordering, and the disordered/segregating Cr and Fe. The strongly ordering and disordered/segregating groups play key roles in the first and second steps, respectively, of the cooling transformation. **b** The *P*_Al-*j*_ indicate that Co and Ni are the strongly preferred neighbors of Al. While the fraction of Fe neighbors is nearly constant above 600 °C, this trend drops off sharply below this temperature. **c** The *P*_Fe-*j*_ show a distinct change near 600 °C, consistent with segregation from the Al-rich matrix. **d** The *P*_Cr*-j*_ show a similar trend as the *P*_Fe*-j*_, except that the Cr-Al-pair probability is lower

The simulations show a rapid change in short-range chemical order for all compositions, at temperatures well below the B2-ordering temperature, demonstrated for Al_2_CoCrFeNi in Fig. 5. While composition changes and phase transformations are expected during cooling, we observe a rapid change over a narrow temperature range, where the weakly ordered Cr/Fe atoms appear to precipitate from the B2 phase into a disordered solid-solution phase, simultaneous with the enhanced ordering behavior of the B2 phase. This trend is exhibited in Fig. 1 for the *x* = 1 compound, where a weakly ordered system at 800 °C clearly is two-phase at 400 °C. The sharpness of this change in behavior for *x* = 2 is evident in Fig. 5, showing the distribution of different pair types. The *P*_*ij*_ continuously change with respect to temperature throughout the transformation, although there is an abrupt change in the trend near 600 °C. This is particularly true for the Fe-Fe and Cr-Cr correlations (Fig. 5a) and other Cr correlations (Fig. 5d). Cr and Fe are identified as disordered because the *P*_Cr-Cr_ and *P*_Fe-Fe_ values are close to the random-mixing value of *x*_Cr_ = *x*_Fe_ = 1/6 at temperatures above 700 °C (Fig. 5a), and at all temperatures shown, *P*_Cr-Fe_ ≈ *P*_Cr-Cr_ and *P*_Fe-Fe_ ≈ *P*_Fe-Cr_, indicating that Cr and Fe continue be to be closely mixed even as these quantities change rapidly below 600 °C. For Cr, above this temperature, the number of Co, Cr, Fe, and Ni pairs are approximately equal (Fig. 5d); below this temperature, the number of Cr-Cr and Cr-Fe pairs rapidly increases, while the number of other Cr-TM pairs rapidly drops. The dramatic increase in both the *P*_Cr-Cr_ and *P*_Fe-Fe_ (Fig. 5a) at temperatures below 600 °C is an indication of phase separation, occurring during cooling. The Al, Co, and Ni elements are identified as ordered because their self-pair probabilities all tend toward zero during cooling (Fig. 5a), and strongly paired with Ni and Co (Fig. 5b). This feature is consistent with the order-parameter analysis in Fig. 3, showing strong ordering of Ni/Co with Al for all temperatures below ~1600 °C (the ordering onset occurs at 1850 °C) for the *x* = 2 composition. This transition behavior is further shown in the pairs of Al, Fe, and Cr pairs, in Fig. 5b–d. Figure 5b shows that above 600 °C, the fraction of Fe-Al pairs is nearly constant. However, cooling below this temperature, the number of pairs drop off dramatically.

As discussed below, the transition near 600 °C is seen for all compositions between *x* = 1 and 2, and is roughly temperature-independent. Similar results are observed for all compositions, as presented in the Supplementary Figures 1 and 2 showing the evolution of pair fractions for Al and Cr for all compositions. Supplementary Figure S2 also shows that the ratio *P*_Cr-Ni_*/P*_Cr-Cr_ changes sharply near 600 °C for all systems, and serves as a strong indicator for this change.

In situ scanning transmission electron microscopy (STEM) and differential scanning calorimetry (DSC) experiments support these observations. The DSC work on AlCoCrFeNi (*x* = 1) shows a peak near 575 °C, in the range of the simulated value of 565 ± 30 °C (see the Supplementary Figure 3). In situ STEM was conducted, while heating an Al_1.3_CoCrCuFeNi alloy from room temperature to 700 °C. This alloy was previously studied using in situ neutron and X-ray diffraction, from room temperature all the way to the melt^10^. As noted earlier and in the literature previously^10^, the Cu phase separates, and does not significantly affect the transitions in the remaining matrix. Only subtle changes were observed in the diffraction patterns between room temperature and 700 °C, which were attributed to the temperature evolution of the highly coherent microstructure dominated by dual BCC/B2 phases. Because the dual phases have nearly the same crystal periodicity, it was recognized that major microstructural transformation may be responsible for the rather small changes in the diffraction signals. The present STEM study provides the direct experimental observation needed to verify these microstructural transformations.

**Fig. 6:**
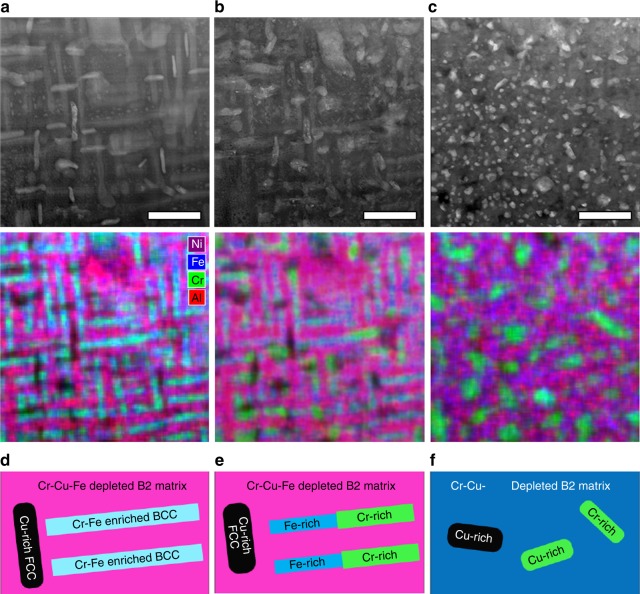
In situ STEM/EDS demonstrate rapid changes in the microstructure between 600 and 700 °C. Representative ADF STEM images and corresponding EDS Maps (Ni, Fe, Cr, and Al) of the Al_1.3_CoCrCuFeNi microstructure. **a** Initial room-temperature microstructure shows an Al-Ni-enriched matrix with Cr-Fe-enriched plates. **b** Heat treatment at 600 °C causes separation of Cr and Fe within the plates. **c** Heat treatment at 700 °C breaks down the coherent microstructure. The observed microstructures are represented schematically in **d** room temperature, **e** 600 °C, and **f** 700 °C. Scale bars are 500 nm

A plate-like microstructure, as found in many Al_*x*_CoCrCuFeNi alloys^10,17,21,26^, is observed in the room-temperature Al_1.3_CoCrCuFeNi micrograph (Fig. 6a), which is color-coded according to abundances of Al (red), Cr (green), Fe (blue), and Ni (magenta) detected from the energy dispersive spectrometry (EDS). The Co and Cu EDS signals are suppressed to more clearly highlight the differences between the matrix, having a reddish appearance, and the blue/green plates, which are enriched in Cr and Fe (Fig. 6a). The Cr-Fe-enriched plates begin to decompose upon heating to 600 °C, such that the Fe and Cr migrate to separate regions within plates, as shown by the nonuniform color (Fig. 6b). Further heating to 700 °C causes a complete breakdown of the plate-like morphology, such that the globular Cr-rich regions are dispersed throughout the matrix, and the Fe appears to have dissolved into the matrix (Fig. 6c). Schematic illustrations (Fig. 6d–f) are shown below the micrographs (Fig. 6a–c) to give a simplified version of the observed microstructure transformation.

Our Monte Carlo simulations exhibit very similar behaviors. The temperature range over which the morphology is observed to change is presented as green bars in Fig. 5. As we see, this feature closely corresponds to the changes observed in the simulated short-range order. The reduced ordering of Fe, compared to Cr, is seen both in the experiment and in the pair behavior of Fig. 5a, where the Fe-Fe pairs are essentially random above 700 °C, while Cr-Cr pairs retain significant order to much higher temperatures. Figure 5b also shows that there is a sharp change in the Fe-Al pairs over this temperature range, while the Cr-Al pair presents a much more gradual change. This feature is consistent with the observed rapid change in behavior of Fe near 600 °C (Fig. 6b, e), and the more gradual change in the Cr-rich regions on heating to 700 °C (Fig. 6c, f). We note that the length scales in our simulations are dramatically different than those on the microscope images, and that these limited length scales may prevent a full description of the behavior. The Supplementary Figures 1 and 2 show that the simulations reveal only a weak dependence of this transformation with the Al content.

**Fig. 7:**
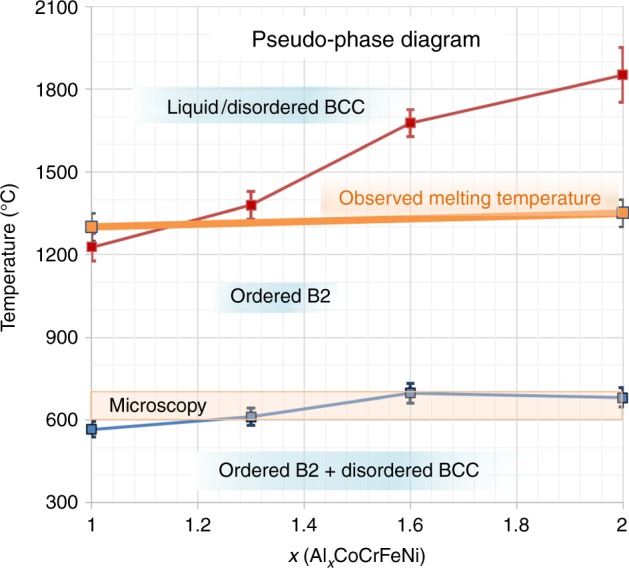
Schematic phase diagram for solid phases of Al_*x*_CoCrFeNi in the range from *1* < x < 2. This diagram captures the simulated trends in B2-ordering temperature with Al content (red data points) and the temperature at which the primarily Cr-Fe BCC phase develops (blue data points). Note that above ~1300 °C, the solid is observed to melt (orange line); thus, for higher Al content, the melt will crystallize into the B2 phase, as observed in scattering experiments presented in Fig. 4. The lower transition is also marked by the 600–700 °C range of temperatures where in situ microscopy demonstrates a change in the microstructure, as shown in Fig. 6

We observe the following trends for Al_*x*_CoCrFeNi, for the range 1 < *x* < 2, summarized in Fig. 7: there is a strongly *x-*dependent B2-ordering temperature, ranging in the simulations from approximately 1220 °C for *x* = 1 to 1850 °C for *x* = 2. For *x* = 1, this value is close to the temperature where B2 superlattice peaks are first observed (Fig. 4b). These compounds melt near 1300 °C (Fig. 4a, b). Thus, for *x* = 2, the prediction is that the compound will have strong B2 order on solidification, consistent with our scattering experiments (Fig. 4a). We predict that the system will have weak B2 order on solidification for most compositions in this range. We further predict a weakly *x-*dependent temperature where the B2 and BCC solid solutions become strongly separated. This unexpectedly sharp transformation occurs very closely to an observed change in the microstructure, using in situ microscopy during heating (Fig. 6b, c), and at a temperature very close to a peak observed through calorimetry for *x* = 1 (Supplementary Figure S3). The observed transition is quite similar to the simulations, with the B2 phase being primarily Ni, Co, and Al (Figs. 2 and 6b), and the disordered phase being primarily Fe and Cr (Fig. 4a, c).

Even though the pathway to phase separation and microstructure development in HEAs may begin with sublattice ordering and coherent phase separation, the equilibrium microstructures sometimes contain phases that are structurally unrelated to the primary solid-solution structures. For example, recent studies using the calculation of phase diagrams (CALPHAD) on the Al_*x*_CoCrFeNi alloys have predicted the formation of sigma phases, which experimentally appear only after hundreds of hours aging at temperatures in the range of 700–1000 °C^22,23^. The as-cast alloys show the simpler microstructures, containing FCC, BCC, and their ordered derivatives^28^. The present simulations focus on the observed BCC-based metastable microstructures, which form during the casting process, and even in many heat-treated alloys, in order to explore the HEA temperature-composition space to identify regions, where interesting and potentially useful transformations occur. The transformations proceed in such a way that the configurational entropy of the overall atomic population decreases during cooling, as expected, although retaining significant disorder in the lattice.

## Discussion

The results presented here represent a remarkably simple theoretical description of multiple transitions occurring in Al-containing HEAs. The Monte Carlo results presented here have no parameters from experiment, derived instead from the enthalpy matrix presented in ref. ^7^, which is based upon high-throughput first-principle calculations. The advantage of the Monte Carlo simulations is that they build in the enthalpies associated with forming ordered phases, while simultaneously capturing the large amounts of disorder (and associated entropy) that occur in each of the phases. These high-throughput simulations are based entirely on nearest-neighbor interactions on a rigid lattice, in the same fashion as the early studies on binary systems^1^, and successfully reproduce major features of the phase evolution observed in Al-containing HEAs, including the B2-ordering behavior at high temperatures, and the B2/BCC separation near 600 °C.

While we expect that interactions beyond near-neighbor pairs would be significant, and furthermore that other thermodynamic considerations (particularly vibrational entropy effects^29,37–40^) that are presently ignored in our model here, the results demonstrate both qualitative and reasonably quantitative comparisons to experiment: scattering provides a key test for B2 ordering (Fig. 4), while the predicted B2/BCC separation near 600 °C (Fig. 5) is supported by in situ microscopy experiments (Fig. 6), and DSC (Supplementary Figure 3). The current approach suggests a broader approach to rapid exploration of phase evolution, where little experimental data exist. The present strategy directly explores the chemical ordering in these “high-entropy” and related complex concentrated alloys, providing an important complement to other important techniques, such as CALPHAD in the quest for developing next-generation materials.

## Methods

### Neutron scattering

Neutrons, having no electric charge, are a highly penetrating probe for studying the structural and dynamic properties of materials^41,42^. Neutron scattering techniques include diffraction, which gives information on the long-range order of crystalline materials, and pair distribution function analysis, which gives information on the local atomic order.

Neutron scattering is well-suited for the present work, because the technique is sensitive to the ordering of the multiple TM elements within HEAs, and the experiments may be conducted using special environments for in situ heating and melting. In the present work, neutron scattering studies were performed at the Oak Ridge National Laboratory, Spallation Neutron Source, using the Nanoscale-ordered Materials Diffractometer^43^. The experiments were conducted over a wide temperature range, from room temperature to 1600°C, using an aerodynamic levitator^10,35,36^. The levitator provides a containerless environment, in which small samples (~2-mm-diameter spheres) are suspended above a conical nozzle with flowing argon gas, and heated with a 250 W CO_2_ laser operating at a wavelength of 10.6 μm. Therefore, the levitator ensures that the phase transformations are not influenced by interactions between the sample and container, allowing in situ neutron studies of the intrinsic transformations. Typical neutron measurement times were 15 min at each temperature, which were started after a few minutes’ equilibration. The heating and temperature regulation procedure was to adjust the laser power, while monitoring the sample temperature using a pyrometer.

### Microscopy

The in situ STEM experiments were performed at the ORNL Center for Nanophase Materials Sciences using a Protochips Aduro in situ heating holder and a Hitachi HF3300 S/TEM operating at 300 kV. The HEA samples were prepared by cutting a small piece (~5 mm cube) from a bulk ingot using standing polishing. Thin, electron transparent specimens were then prepared using a Hitachi NB5000 focused ion beam (FIB) technique. The FIB-extracted specimen was then mounted to a resistive-heating chip, and placed in the S/TEM for examination at a series of temperatures, starting with ambient conditions. STEM images were collected at controlled elevated temperatures. The EDS data were collected using a Bruker silicon drift EDS detector after quenching to room temperature. Thus, the EDS measurements were not conducted at high temperatures. However, all STEM images were collected in situ at the reported temperatures. Note that the EDS and STEM images (Fig. 6) match well, indicating that no significant microstructural changes occurred due to temperature quenching between measurements.

### Monte Carlo simulations

Monte Carlo simulations use random numbers to generate events, such as the jumping of atoms between lattice sites, which are accepted or rejected according to probabilities based upon physical models^44^. Here the mixing behavior of an HEA is simulated by populating a truncated lattice with multiple elements (two to six different elements), initially in a random configuration, or, alternately, in a sequence where each element fills a contiguous block of sites, representing an “unmixed” state. The simulation proceeds by allowing randomly selected atom pairs to exchange positions, or jump. The jumps are accepted or rejected according to a Boltzmann probability, *P*, given by

3 $$P = {\mathrm{exp}}\frac{{ - {\mathrm{\Delta }}H}}{{k_{\mathrm{B}}T}},$$

where Δ*H* is the enthalpy change caused by the jump, *k*_B_ is the Boltzmann constant, and *T* is the temperature.

Each atom on the B2 lattice has eight nearest neighbors, and Δ*H* may be approximated, using the same binary interaction parameters, *v*_*ij*_, as in the Bragg-Williams model^1^. In contrast to the Bragg-Williams model, however, the Monte Carlo simulation evaluates v_*ij*_ for discrete atom pairs, throughout the lattice. Furthermore, the long-range order parameters, *η*_*i*_, which are the required inputs for solving the Bragg-Williams expressions, may be extracted from the Monte Carlo simulations, without any prior knowledge of the ordering trends.

Consider two lattice sites, *L* and *M*, which are occupied by elements of types, *l* and *m*, respectively, and are surrounded by nearest-neighbor atoms of types, *l*(*k*) and *m*(*k*), respectively, where *k* = 1–8 for the BCC structure. If the atoms on sites, *L* and *M*, jump and trade places, then the Δ*H* used in the Boltzmann probability of Eq. (3) may be written as

4 $${\mathrm{\Delta }}H_{{\mathrm{jump}}} = \mathop {\sum}\limits_{k = 1}^8 {\left[ {\left( {v_{l,m(k)} + v_{m,l(k)}} \right) - \left( {v_{l,l(k)} + v_{m,m(k)}} \right)} \right]}$$

The jump is always accepted, when Δ*H*_jump_ is negative, but accepted only by “chance” when Δ*H*_jump_ is positive, according to the probability, *P*, given by Eq. (3). As the Monte Carlo simulation is run for thousands of jumps, the sum of the Δ*H*_jump_ for all accepted jumps asymptotically approaches an equilibrium value, as the atoms reach an equilibrium configuration.

The long-range order parameters, *η*_*i*_, were extracted from the simulation simply by comparing the elemental populations on each sublattice. In particular,

5 $$\eta _i\left( {{\mathrm{simulation}}} \right) = \frac{{N_{i\alpha } - N_{i{\mathrm{Av}}}}}{{N_{i{\mathrm{Av}}}}}$$

where *N*_*iα*_ is the number of type-*i* atoms on the α sublattice, and *N*_*i*Av_ is the average number of type-*i* atoms on each sublattice. Thus, the Monte Carlo simulation may inform the choice of *η*_*i*_, used in the above described Bragg-Williams calculations.

### Differential scanning calorimetry

The solidification temperature and possible solid-solid phase transition temperature of the AlCoCrNiFe (*x* = 1) equiatomic HEA were measured using a NETZSCH 404C differential scanning calorimeter. The measurement was run at a constant cool rate of 20 C/min from 1450 to 250 °C under a flowing argon atmosphere. Results are shown in Supplementary Figure 3.

### Code availability

The Metropolis Monte Carlo used in this study was developed by L.J.S., for use within MATLAB. This code is available on request from Santodonato (lsantod1@vols.utk.edu) or the corresponding author J.R.M. (morrisj@ornl.gov).

## Electronic supplementary material

Supplementary Information

## Data Availability

All figures have associated raw data, available on request from the corresponding author, J.R.M. (morrisj@ornl.gov), or from the first author, L.J.S. (lsantod1@vols.utk.edu).
