# Supplementary material for: Predictive multiphase evolution in Al-containing high-entropy alloys
Source: Nat Commun. 2018 Oct 30;9:4520. doi: 10.1038/s41467-018-06757-2 (PMC6207727; doi:10.1038/s41467-018-06757-2)
Supplement: Supplementary file 1 — Supplementary Information [file 41467_2018_6757_MOESM1_ESM.pdf]

Predictive multiphase evolution in Al-containing high-entropy alloys

L. J. Santodonato et al.

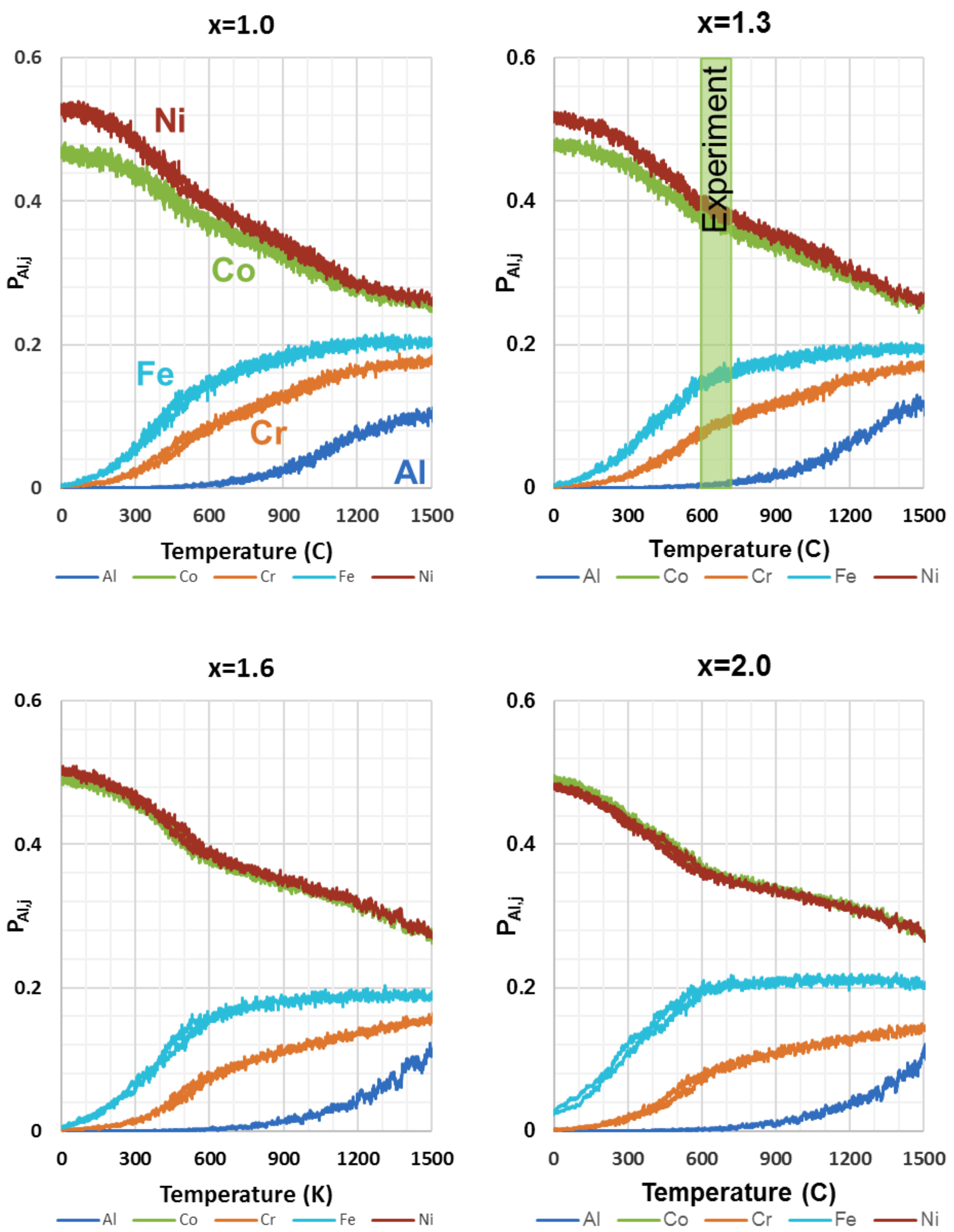

Supplementary Figure 1. Al pair correlations for Al$_x$CoCrFeNi in the range from $1 < x < 2$.

These plots show the fractions of neighbor types for Al atoms, as a function of composition and temperature. Note that the Al strongly favors Ni and Co at all temperatures. As the Al content increases, the fraction of Al-Al pairs actually decreases, due to the stronger tendency toward the B2 formation. The bar labelled "Experiment" for x = 1.3 indicates the range of temperatures where the Co/Fe-rich BCC phase is observed to dissolve, as shown in Fig. 6. The change in the Al-Fe pair behavior near this temperature range is clear for all compositions.

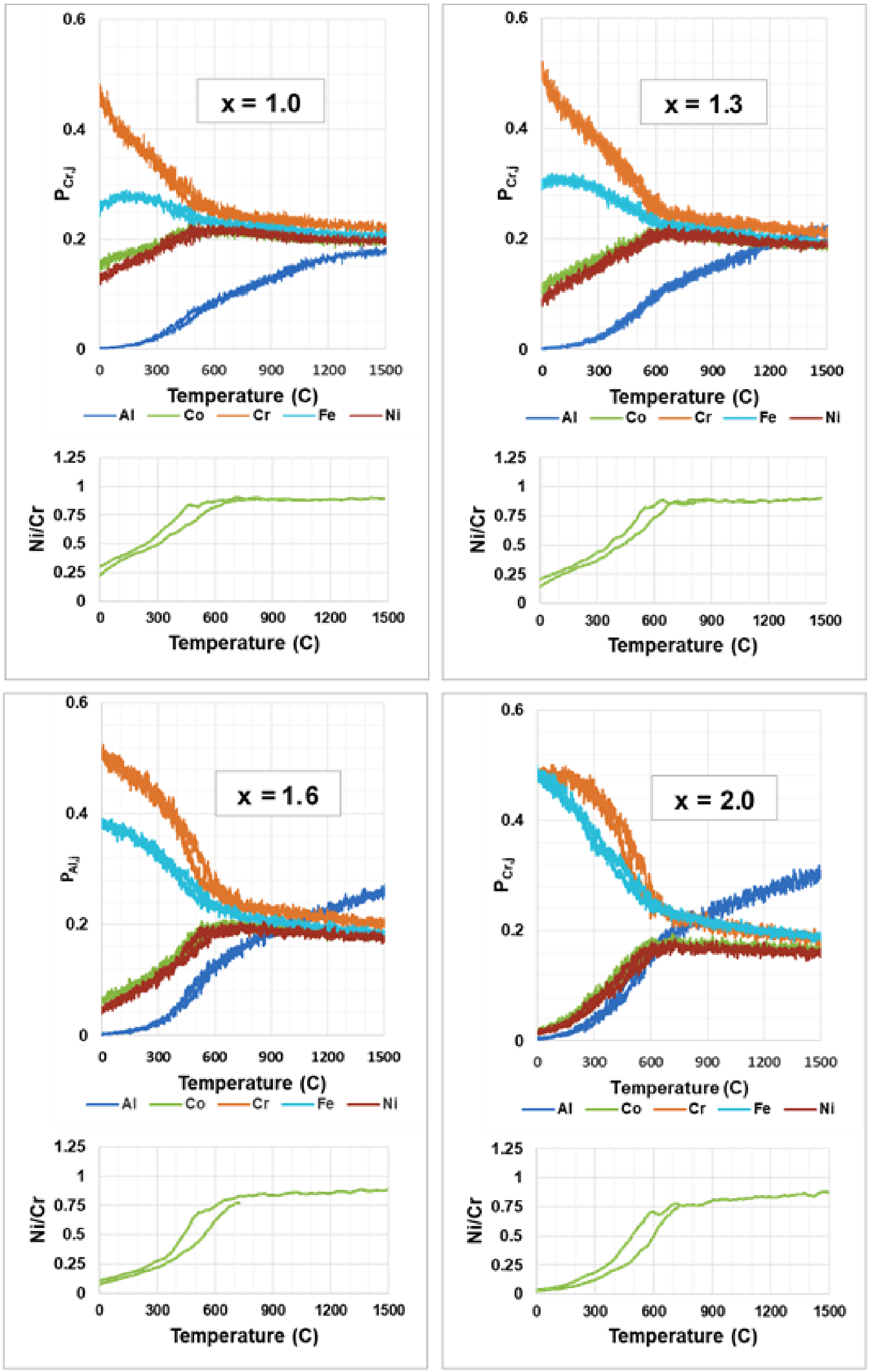

Supplementary Figure 2. Cr pair correlations for Al_xCoCrFeNi in the range from $1 < x < 2$.

Fractions of neighbor types for Cr atoms, and ratio of Cr-Ni to Cr-Cr pairs, for each composition. In the range 600-700 °C, the Co/Fe-rich BCC phase is observed to dissolve, as presented in Fig. 6. Above this temperature, Cr has approximately equal neighbors of any of the TM. Below this temperature, the fraction of pairs changes significantly, with Cr favoring Cr and Fe (consistent with the results shown in Fig. 6).

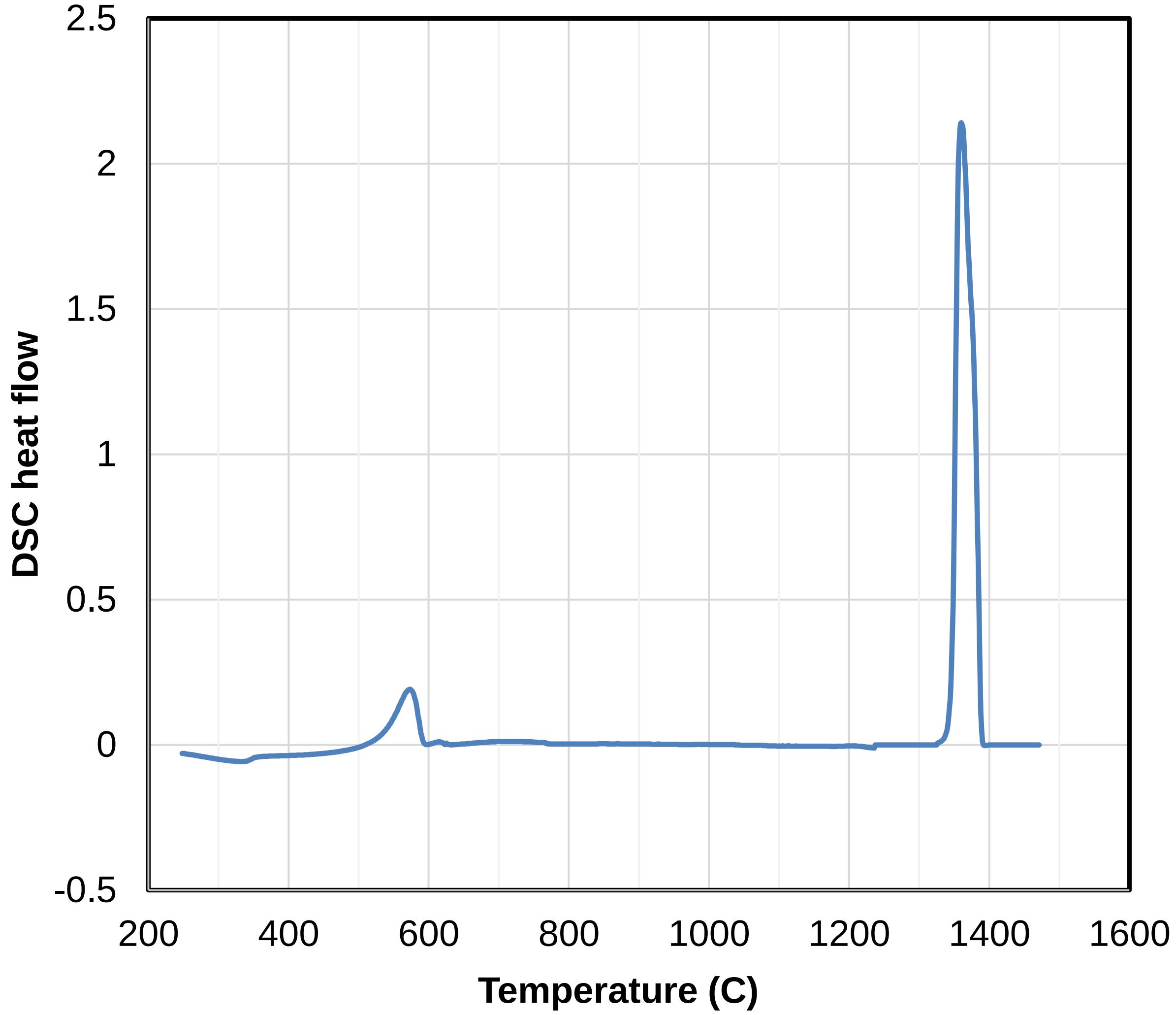

Supplementary Figure 3. Differential scanning calorimetry (DSC) measurement for AlCoCrFeNi.

DSC for AlCoCrFeNi shows a sharp peak at the melting temperature of 1,360 °C, and a second peak near 575 °C, in the range of the simulated value of 565 ± 30 °C indicated in Fig. 7.
